# Single-cell analysis reveals key differences between early-stage and late-stage systemic sclerosis skin across autoantibody subgroups

**DOI:** 10.1136/ard-2023-224184

**Published:** 2023-08-14

**Authors:** Kristina Elizabeth Neergaard Clark, Shiwen Xu, Moustafa Attah, Voon H Ong, Christopher Dominic Buckley, Christopher P Denton

**Affiliations:** 1 Centre for Rheumatology, Royal Free Campus, University College London, London, UK; 2 Kennedy Institute of Rheumatology, University of Oxford, Oxford, UK

**Keywords:** Scleroderma, Systemic, Fibroblasts, Autoantibodies

## Abstract

**Objectives:**

The severity of skin involvement in diffuse cutaneous systemic sclerosis (dcSSc) depends on stage of disease and differs between anti-RNA-polymerase III (ARA) and anti-topoisomerase antibody (ATA) subsets. We have investigated cellular differences in well-characterised dcSSc patients compared with healthy controls (HCs).

**Methods:**

We performed single-cell RNA sequencing on 4 mm skin biopsy samples from 12 patients with dcSSc and HCs (n=3) using droplet-based sequencing (10× genomics). Patients were well characterised by stage (>5 or <5 years disease duration) and autoantibody (ATA+ or ARA+). Analysis of whole skin cell subsets and fibroblast subpopulations across stage and ANA subgroup were used to interpret potential cellular differences anchored by these subgroups.

**Results:**

Fifteen forearm skin biopsies were analysed. There was a clear separation of SSc samples, by disease, stage and antibody, for all cells and fibroblast subclusters. Further analysis revealed differing cell cluster gene expression profiles between ATA+ and ARA+ patients. Cell-to-cell interaction suggest differing interactions between early and late stages of disease and autoantibody. TGFβ response was mainly seen in fibroblasts and smooth muscle cells in early ATA+dcSSc skin samples, whereas in early ARA+dcSSc patient skin samples, the responding cells were endothelial, reflect broader differences between clinical phenotypes and distinct skin score trajectories across autoantibody subgroups of dcSSc.

**Conclusions:**

We have identified cellular differences between the two main autoantibody subsets in dcSSc (ARA+ and ATA+). These differences reinforce the importance of considering autoantibody and stage of disease in management and trial design in SSc.

**WHAT IS ALREADY KNOWN ON THIS TOPIC**

- Skin fibrosis in diffuse systemic sclerosis (SSc) generally improves in late disease with trajectory of change differing across antinuclear antibody subgroups.
- Anti-RNA polymerase (ARA) positive patients generally improve and stabilise skin more than anti-topoisomerase-1 (ATA) positive.
- Cellular mechanisms underlying distinct trajectories will give insight into the drivers of improvement in SSc.

**WHAT THIS STUDY ADDS**

- This study demonstrates a cellular basis for the differences in skin severity between ATA+ and ARA+ early-stage and late-stage diffuse cutaneous SSc (dcSSc).
- Single-cell gene expression between stages and antibody subset of disease with notable differences in ligand receptor interactions between key stromal cells within the skin.

**HOW THIS STUDY MIGHT AFFECT RESEARCH, PRACTICE OR POLICY**

- Our study shows the importance of considering disease stage and autoantibody when treating dcSSc and designing clinical trials to improve response to treatment targeting a particular cell type or cytokine pathway.

## Introduction

The extent and severity of skin thickness varies in systemic sclerosis (SSc). In diffuse cutaneous SSc (dcSSc), skin thickness correlates with clinical outcome including survival and risk of internal organ complications.^1^ Skin severity worsens in early dcSSc, then often plateaus or improves later.^2^ It is notable that the development of new cardiac, pulmonary or renal involvement is much less frequent at later stages but may be more serious when severe skin thickening persists.^2^

Two disease-specific antinuclear antibody (ANA) specificities in dcSSc are anti-topoisomerase-1 (ATA) and anti-RNA polymerase III (ARA). Clinical phenotype in SSc is linked to the pattern of SSc hallmark autoantibodies. For example, patients with ATA have high risk of developing lung fibrosis regardless of the extent of skin involvement, whereas greatest risk of renal crisis is for patients with ARA.^1^ Skin severity and skin score trajectory differ between autoantibody groups. Thus, ARA associates with higher peak mRSS, but greater improvement in mRSS over time, so that cases are typically more severe in early-stage disease but much less affected in later disease.^3^ This capacity for improvement at least partly reflects natural history as it is observed in the placebo arm of controlled trials without background immunosuppression. It is plausible that differences in trajectory of improvement have a biological basis and that studying the cellular and molecular differences across a group of well-characterised patients with early-stage or late-stage dcSSc that differ between ARA and ATA offers a platform to elucidate some of the likely pathogenic differences that lead to distinct patterns of skin fibrosis.^1 4–6^ Despite evidence linking ANA reactivity to different clinical manifestations,^1 7^ and underlying differences in molecular phenotypes,^8^ there is a limited evidence for a pathogenic role of SSc-specific autoantibodies, with only ATA potentially modulating fibroblasts and endothelial cells in vitro.^9 10^

This study addresses the cellular basis for differences in the stage of skin disease, and autoantibody status by performing detailed single-cell transcriptomic analysis of well characterised cases of dcSSc. We explore the specific hypothesis that intrinsic differences between fibroblast populations, and their interactions with other cell clusters, may reflect clinical diversity of skin in SSc.

## Methods

This was a single-centre observational study of five distinct cohorts, such as: early ATA+ or ARA+dcSSc (<5 years disease duration), established ATA+ or ARA+dcSSc and healthy controls (HCs). In total 15 participants were recruited (3 per cohort). Recruitment occurred in parallel.

### Patient and public involvement

All subjects provided 4 mm skin biopsies, following informed consent for their inclusion in the study, and for the use of their clinical data and samples for research purposes.

Patients with SSc all fulfilled the 2013 American College of Rheumatology/European League against Rheumatism classification criteria,^11^ and only patients with a skin distribution consistent with dcSSc according to^12 12^ were included. Clinical information collected included autoantibody status, disease duration and mRSS, which was assessed at the time of sample collection, and all measurements by one assessor.

### Sample collection

Paired 4 mm skin biopsies were obtained from the forearm of subjects, and initially placed in MACS Tissue storage solution (Miltenyi Biotech Inc), for transfer. Paired samples were processed for histological examination.

### Skin dissociation technique

Preliminary work in our laboratory (data not shown) and published work^13^ support the comparable number, and gene expression of fresh and frozen skin samples. Therefore, samples were dissociated prior to freezing.

Subcutaneous fat was removed from each skin sample. The sample was dissociated using the Human Whole Skin Dissociation Kit (Miltenyi Biotech Inc) with Enzyme P, with overnight incubation as per manufacturers guidelines. The dissociated cells were stored in CryoStor CS10 (StemCell Technologies) at −80°C, then transferred to liquid nitrogen after 24 hours.

### Thawing

Thawing occurred just prior to 7-AAD staining (BioLegend 420404) and viable cell sorting by the SH800 Cell sorter (Sony Biotechnology). Viable cells in single-cell suspension were resuspended in 1% BSA in phosphate-buffered saline (PBS) at a concentration of 1000 cells/µL. A maximum of 20 000 cells were counted using the fluorescence cell counter LUNA-FX7 (Logos Biosystems) and loaded onto a single 10× lane and processed with the 10× Genomics Single Cell 3’ kit (V.3.1) following manufacturer user guide (CG000330). Only 3 samples did not reach this target, and about 17 000 cells were loaded. Sequencing was carried out by the Oxford Genomics Centre, using Illumina NovaSeq 6000 (V.1.5 chemistry, 28 bp/98 bp) and libraries were sequenced to a minimum of 50 000 reads/cell.

Cells were analysed in two batches. Four samples were run initially, and included samples from across the subgroup spectrum, and subsequently all remaining cells were run in a second batch.

FASTQ files were demultiplexed for each 10× library using the Cell Ranger (V.3.1.0) mkfastq function. Reads were mapped to the GRCh38 human genome.

### Statistical analysis

Statistical analysis was carried out in R software (V.4.0.2), using the Rpackage ‘Seurat’ (V.4.2.0). Integration was performed using Harmony. Cell clusters were identified using top markers and referencing the Human protein atlas (https://www.proteinatlas.org/). Subsetting for fibroblasts was performed on all samples, and reclustering was performed following the Seurat pipeline.

Pseudobulk with the packages ‘tidyverse’, ‘edgeR’, ‘SingleCellExperiment’ and ‘DESeq2’ was used for PCA construction.

KEGG pathway analysis was carried out using the package ‘gsfisher’. Further gene set enrichment for was carried out using ‘fgsea’.

Volcano plots were created using ‘EnhancedVolcano’ package. Trajectory analysis (pseudotime) was performed using packages ‘slingshot’, ‘TSCAN’ and the package ‘CellChat’ was used for ligand receptor interactions.

## Results

### Study cohort

Fifteen forearm dermal punch biopsy samples were collected from 12 dcSSc patients and 3 HCs. The median age of SSc patients was 58.1 years (IQR 50.9–69.4). Median disease duration was 87 months (IQR 44–221 months) (table 1). Most participants were female (80%). Patient subgroups were split into early-stage or late-stage disease, with median (IQR) disease duration 2.75 years (37–51 months) and 17.5 years (133–248 months), respectively. There were equal ATA+ and ARA+ patients in each cohort.

**Table 1:** Participant demographics

	Early dcSSc (n=6)	Late dcSSc (n=6)	HC (n=3)
Age (years)	52.8 (45.3–57)	69.4 (60.3–69.9)	42.1 (27.7–47.8)
Female (%)	5 (83.3)	4 (66.7)	3 (100)
Disease duration (mths)	44 (37–51)	221 (133–248)	
mRSS	19.5 (17–22)	7.5 (2–11)	
Antibody			
ARA (%)	3 (50)	3 (50)	
ATA (%)	3 (50)	3 (50)	
Immunosuppression			
MMF (%)	6 (100)	4 (66.7)	
MTX (%)	1 (16.7)	0	
Prednisolone <10 mg (%)	1 (16.7)	2 (33.3)	
Organ complications			
ILD (%)	3 (50)	4 (66.7)	
Myositis (%)	1 (16.7)	0	
Renal crisis (%)	0	1 (16.7)	
PAH (%)	0	1 (16.7)	

Demographics of patients included in the study. Median and IQR reported unless otherwise stated.

ARA, anti RNA polymerase III antibody; ATA, anti-topoisomerase antibody; HC, healthy control; ILD, interstitial lung disease; MMF, mycophenolate mofetil; mRSS, modified Rodnan skin score; MTX, methotrexate; PAH, pulmonary arterial hypertension.

### Whole skin analysis

**Figure 1:**
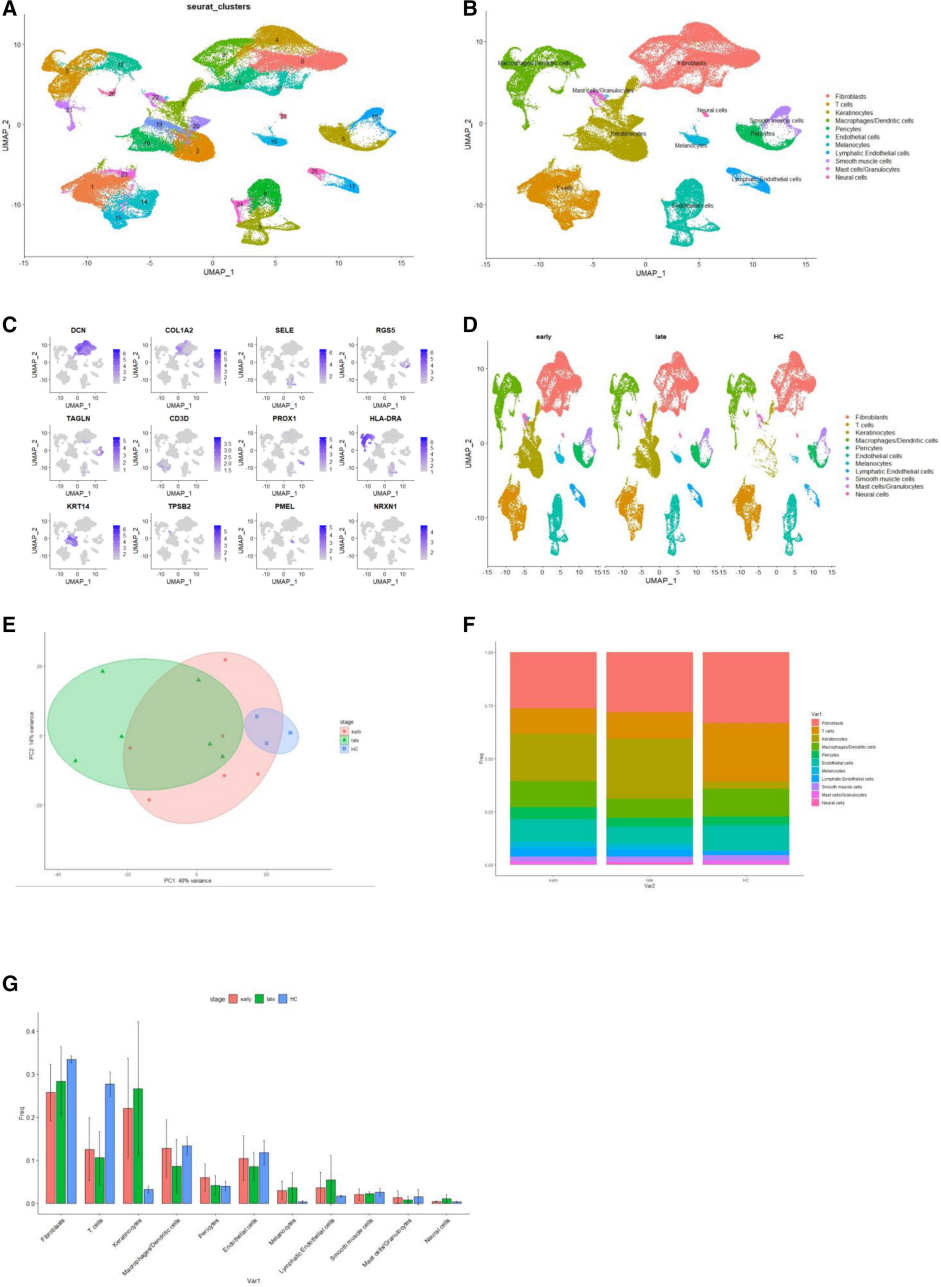
Overview of scRNAseq landscape. Markers to identify clusters, and differences between early-stage and late-stage SSc and HC (A) UMAP of all samples from skin samples. (B) UMAP with named clusters. (C) Feature plot with key genes used to identify clusters. (D) Split UMAP showing gross differences in abundance between early dcSSc, late dcSSc and HC. Most obvious differences apparent between keratinocyte clusters and fibroblast clusters. (E) PCA plot composed using pseudobulk analysis of all cells and all samples, with ellipses highlighting early dcSSc (red), late dcSSc (green) and HC (blue). (F) Bar plot of proportion abundance of each cluster by stage of disease. Notable differences can be seen with a contracted proportion of T cells and expanded keratinocytes in dcSSc. (G) Bar plots showing proportion each cell type by stage of disease red=early dcSSc, green=late dcSSc, blue=HC. dcSSc, diffuse cutaneous systemic sclerosis; HC, healthy control; PCA, principal component analysis; scRNAseq, single-cell RNA sequencing.

The main focus of this study was single-cell RNA sequencing (scRNAseq) analysis. All samples were run through the cellRanger, and filtered genes were integrated with batch correction using Seurat. In total, 124 735 cells were analysed. Clustering identified 28 clusters in total (figure 1A). Marker genes were used to broadly annotate clusters (figure 1B), and genes used to aid identification are shown in figure 1C. Reported marker genes were universally detected across cell types, all scRNAseq assay schemes, all cohorts and most participants. There were gross differences in cluster abundances by disease stage (figure 1D,E), with expansion of keratinocytes in disease subpopulations, and contraction of T cell populations. There was clustering of HC samples, but separation of early and late SSc on pseudobulk PCA. Within the keratinocyte clusters, there was differing gene expression between the early and late dcSSc which included increased expression of ADAMTS1, and EREG (an epidermal growth factor) as disease duration increased (online supplemental figure 1). Complementary histological analysis showed typical features of increased eosinophils in the extracellular matrix (ECM), more densely packed in early-stage SSc skin with inflammatory infiltrates. These features were more prominent in ARA+ biopsies. Inflammation had resolved in later-stage SSc with less dense ECM more similar to HC skin (online supplemental figure 2).

10.1136/ard-2023-224184.supp1 Supplementary data

### Fibroblast cell cluster analysis

**Figure 2:**
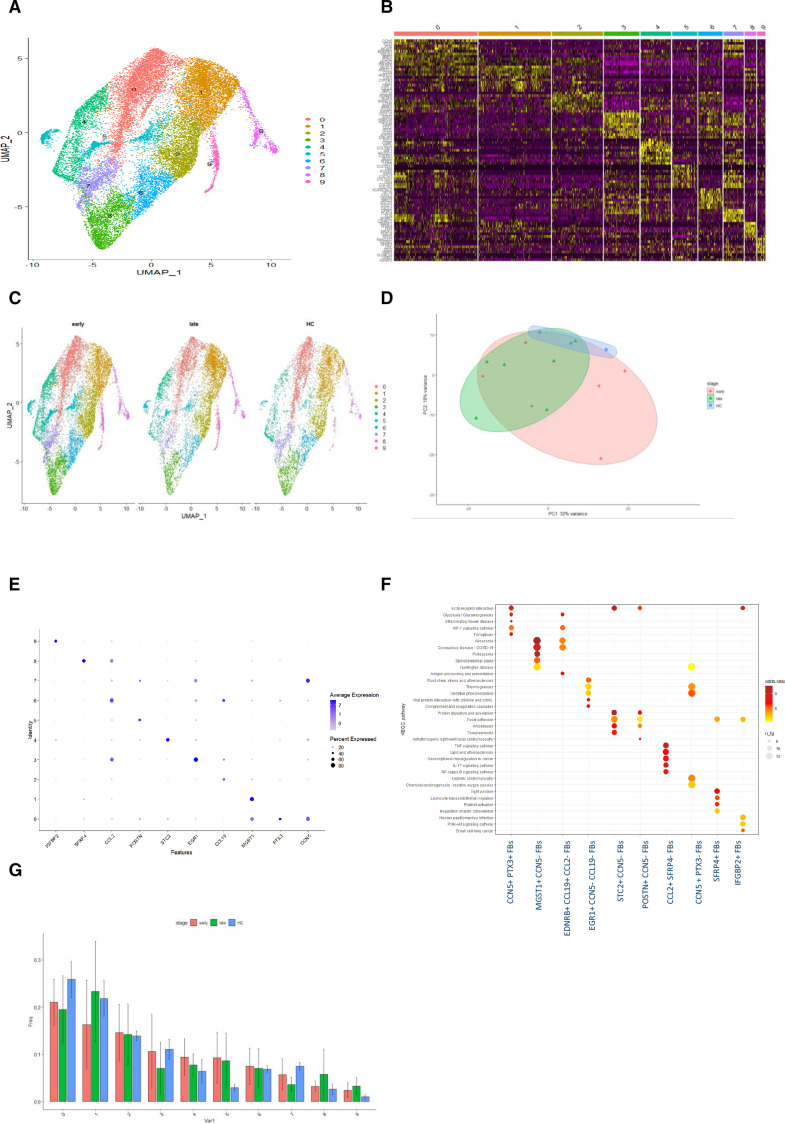
Re-clustered fibroblast landscape for early-stage and late-stage SSc and HC. Heatmap of differentiating genes, differences KEGG pathways and naming fibroblast subsets. (A) UMAP of fibroblast subset from all samples discriminating 10 distinct fibroblast populations. (B) Heatmap of the top 10 differential overexpressed genes by statistical significance for each cluster. (C) Split UMAP of fibroblast clusters by stage of disease. Visually clear differences in cluster 0, cluster 4 and cluster 5. (D) PCA plot from pseudobulk analysis of all fibroblast cells from all samples, with ellipses highlighting early dcSSc (red), late dcSSc (green) and HC (blue). (E) Key differentiating genes by each fibroblast cluster. (F) KEGG pathway analysis, showing clear different gene set enrichment in each fibroblast cluster. (G) Barplot by abundance per subset of fibroblast clusters. dcSSc, diffuse cutaneous systemic sclerosis; HC, healthy control; PCA, principal component analysis.

We next focused on fibroblasts. Reclustering identified 10 fibroblasts clusters (figure 2A). The top five differentiating markers for each cluster are shown in figure 2B. Differences in cell abundance by stage of disease were notable (figure 2C,G). PCA showed clear differentiation between HC clusters and SSc fibroblasts (figure 2D). Cluster 8 had a high expression of SFRP4, and some expression of ACTA2, making it most consistent with a myofibroblast profile. The most abundant fibroblast cluster (cluster zero), had a high expression of CCN5+PTX3+. Cluster 1 had high gene expression for MGST1+, with no expression of CCN5 (CCN5−). Further gene set enrichment analysis of these clusters highlighted differential KEGG pathway expression, with the CCN5+PTX3+ fibroblast cluster showing significant expression of genes associated with ECM-expression interaction, which was not universal across all fibroblast clusters. There was increased expression of HIF-1 signalling pathway and glycolysis/gluconeogenesis in cluster 0. The focal adhesion pathway was most highly expressed in the STC2+CCN5 FB cluster, whereas the IL-17 pathway and the NF-kappa B signalling pathway were overexpressed in the CCL2+SFRP FB cluster (figure 2F).

### Comparison of autoantibody subgroups

**Figure 3:**
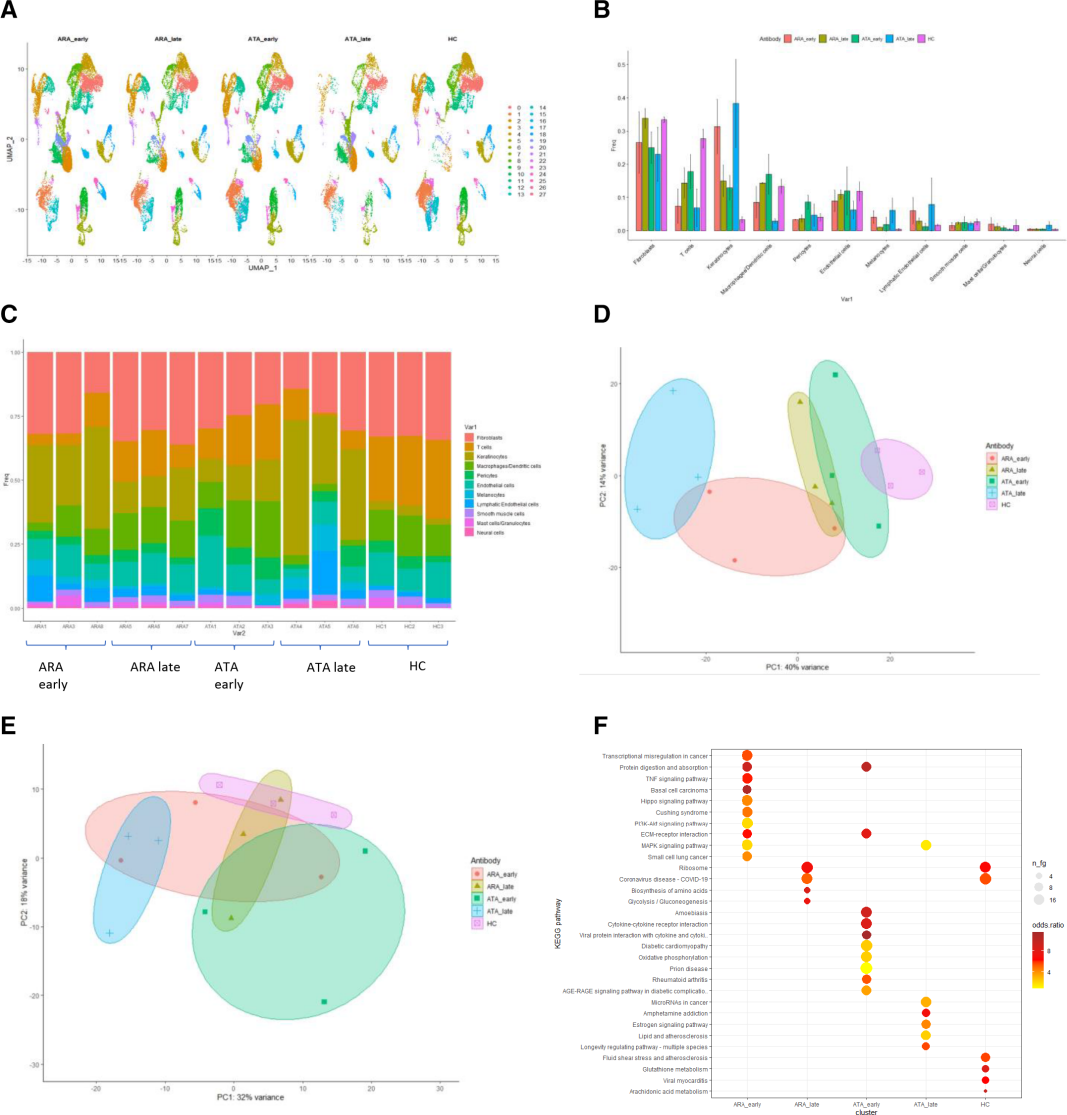
Differences by autoantibody across all cells and within the fibroblast cluster. (A) UMAP plot of whole skin split by stage and autoantibody. (B) bar plot showing mean frequency and SD of each cluster by SSc stage and ANA subset. red=early ARA+dcSSc, olive=late ARA+dcSSc, green=early ATA+dcSSc, blue=late ATA+dcSSC, purple=HC. (C) Stacked bar plot of proportion abundance by individual sample. (D) PCA plot from pseudobulk analysis from whole skin. This shows much clearer differentiation of sample groups when both stage and antibody are taken into consideration. red=early ARA+dcSSc, olive=late ARA+dcSSc, green=early ATA+dcSSc, blue=late ATA+dcSSc, purple=HC. (E) PCA plot from pseudobulk analysis of fibroblast subset. Once again, there is clearer differentiation between the subsets when both stage and antibody are taken into consideration compared with only stage. More marked differentiation is apparent between early and late ATA+, than for ARA+ patients. Red=early ARA+dcSSc, olive=late ARA+dcSSc, green=early ATA+dcSSc, blue=late ATA+dcSSC, purple=HC. (F) KEGG pathway analysis of all fibroblasts by antibody and stage. ARA, anti-RNA-polymerase III; ATA, antitopoisomerase antibody; dcSSc, diffuse cutaneous systemic sclerosis; HC, healthy control; PCA, principal component analysis.

Based on our earlier work,^8^ we next asked whether taking the antibody into account would allow for improved differentiation between subjects, and support our hypothesis that cellular differences between autoantibody subgroups underpin clinical heterogeneity for skin in dcSSc. There were clear differences in cell abundance across populations that were more apparent when the patient populations were split by autoantibody and stage, than by stage alone (figure 3A–C). Principal component analysis (PCA) plots confirmed more distinct clustering of subjects when both characteristics are considered (figure 3D), more pronounced in the ATA patient subset compared with ARA subjects.

There were some differences between the immune clusters by autoantibody (online supplemental figure 3). Specifically, the CD4 T cells were most prominent in the late ATA+dcSSc patients, whereas the NK cells were more predominant in early dcSSc patients compared with late stage. The ATA subgroup showed more variation in macrophage clusters over time compared with the ARA subgroup.

Fibroblast subclustering also gave clearer demarcation of patients when autoantibody and stage were considered, and this was most prominent in the ATA compared with ARA patients (figure 3E and online supplemental figure 4). KEGG pathway analysis across fibroblast clusters shows differences reflecting stage and antibody (figure 3F). Early-stage ARA fibroblasts are dominated by gene pathways relating to HIPPO signalling and PI3-AKTsignaling, whereas early ATA fibroblasts have genes associated with cytokine–cytokine interaction, including the TGFβ family, and cytokines interacting with the IL6ST receptor. Both early-stage subsets show increased pathway expression of ECM-receptor interaction not seen in later-stage SSc.

**Figure 4:**
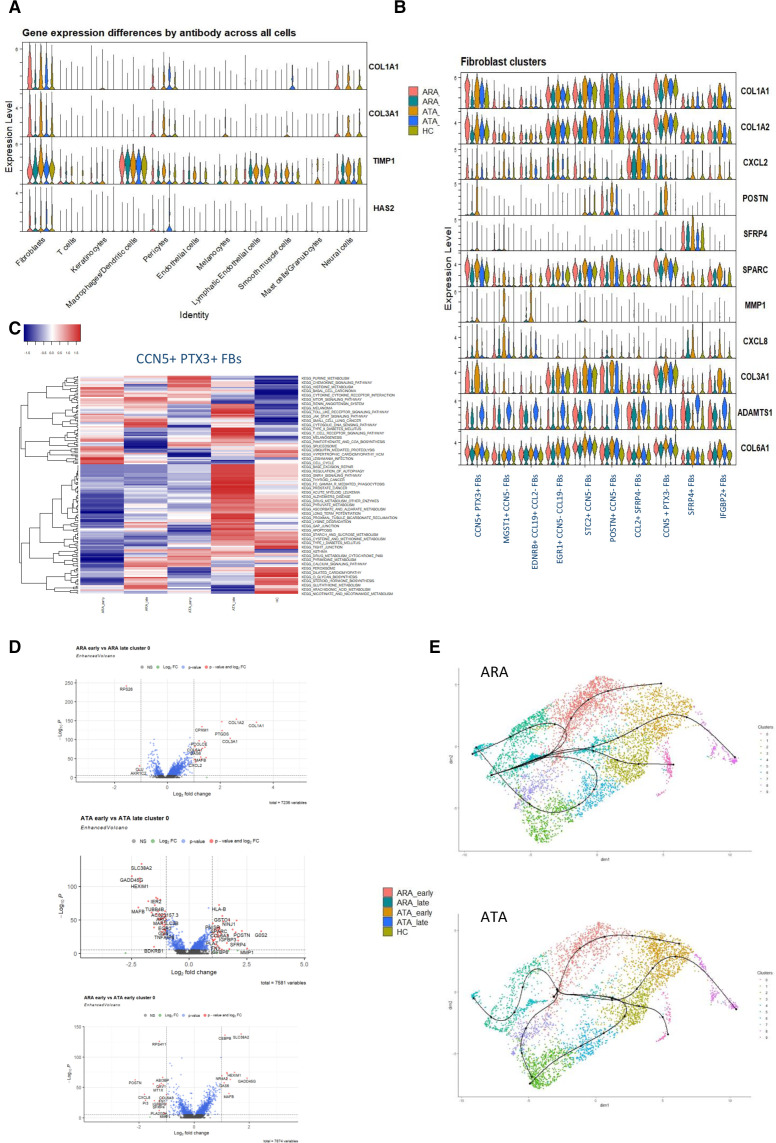
Differences by autoantibody and stage in cluster 0, KEGG differences, key gene expression violin plots and trajectory differences by antibody. (A) Gene expression differences across whole skin of key genes previously identified as having differential protein concentrations in the serum by stage and antibody. (B) Differential expression of key genes within the 10 fibroblast clusters by stage and antibody. Some key profibrotic genes are clearly only expressed in early dcSSc, or in certain clusters. SFRP4 only expressed cluster 8, consistent with myofibroblasts. (C) KEGG pathway differential expression in cluster 0 fibroblasts (CCN5+PTX3+ FBs) between each antibody and stage. (D) Violin plots from cluster 0 fibroblasts (CCN5+, PTX3+). Comparison between ARA+ early and late stage, where most overexpressed genes are seen in early ARA+. ATA+ cluster 0 fibroblasts (CCN5+PTX3+) shows significant differential expression in both earl-stage and late-stage disease. A key set of differentially expressed genes separate FB cluster 0 between early ARA+ and ATA+. (E) Pseudotime analysis of fibroblast clusters. Originator FB cluster in both early antibody subsets was identified as being cluster 0, and myofibroblasts were identified as cluster 8. However, branch points differ between ARA+ and ATA+ early dcSSc, with some terminal fibroblasts being cluster three in ATA+ early dcSSc, whereas in ARA+ early dcSSc, FBs do not terminally differentiate at cluster 3, but include cluster 5 and cluster 4. ARA, anti-RNA-polymerase III; ATA, antitopoisomerase antibody; dcSSc, diffuse cutaneous systemic sclerosis; HC, healthy control.

We have previously reported significant autoantibody associated differences over time in serum levels of TIMP1, PIIINP and HA in early dcSSc.^8^ These analytes correlate with skin score in cross-sectional studies both individually and as part of the composite ELF score.^14 15^ Using scRNAseq analysis, we identify which cells drive altered gene expression (figure 4A). COL1A1 (previously shown to differentiate improvers from progressors^8^) and COL3A1 showed highest expression in early SSc, originating predominantly from the fibroblasts. Increased expression of TIMP1 originates from fibroblasts and monocytes, across all stages of disease, but autoantibody differences were more apparent in the lymphatic endothelial cells, smooth muscle cells and pericytes where expression was higher in the ATA+ patients compared with ARA+.

We next selected some differentiating genes from our earlier work, and candidate profibrotic genes. This highlighted that differences are not restricted to the most abundant fibroblast cluster (figure 4B). Some gene expression variation is consistent across all fibroblast populations (ADAMTS1, COL6A1), whereas for other genes, expression is higher in early compared with late-stage SSc (COL1A1, COL3A1, SPARC). Certain genes seem to be restricted to specific fibroblast clusters of one autoantibody subtype, at a specific stage of the disease (MMP1, POSTN, CXCL8), suggesting that some fibroblast subpopulations are both stage and antibody specific.

Figure 4D highlights the differential expression of genes by stage and autoantibody status. Focusing on the most abundant fibroblast cluster, CCN5+PTX3+ FBs and comparing only ARA patients, there is a clear set of ‘activation genes’ in early-stage SSc, with fewer overexpressed genes during the later stage disease. This pattern, where the majority of overexpressed genes are in early compared with late-stage ARA patients, is also seen in other FB clusters (online supplemental figure 5). However, in contrast, in ATA patients, there is more stage-specific differential gene expression. This may suggest activated genes are not ‘switching off’ in later ATA disease with an ongoing active phenotype, including genes such as EGR3 and TNFAIB in later stage of disease. This pattern of ongoing activity in the later stages of the disease is consistent across all fibroblast clusters of ATA patients (online supplemental figure 5). Lastly, direct comparison between early ARA and early ATA patients in the CCN5+PTX3+ fibroblast cluster highlights unique autoantibody differences in early-stage SSc, which may start to explain some clinical differences in disease phenotype. Key fibrosis associated genes such as POSTN, SFRP4 are more abundant in early ATA+ patients compared with early ARA+ patients. This is further supported by KEGG pathway analysis by stage and antibody subset across all samples (figure 4C). There are clear similarities between ARA early and ATA early samples with upregulation of pathways including cytokine–cytokine interaction, chemokine signalling. However, unique pathways are seen upregulated in the ARA early patients such as T cell receptor signalling, and JAK STAT signalling more than is seen in ATA early patients, whereas alternative pathways are unique to the ATA early dcSSc patients such as calcium signalling pathway. There is more similarity between ARA late patients and HC pathway activation, than ATA late SSc, which supports the idea of an ongoing active fibroblast phenotype in the ATA compared with the ARA late-stage patients.

Trajectory analysis in early dcSSc confirms differences between the autoantibody subtypes (figure 4E). Entropy analysis of gene expression supports cluster 0 being an early-stage fibroblast, and cluster 8 is likely an end point cluster based on high SFRP4+ expression, and ACTA2+ expression.^16^ Using these anchor time points, we used this technique to highlight differences in the relationship between the fibroblast subsets based on autoantibody. Although both autoantibody subtypes show a trajectory which leads from cluster 0 to cluster 8, there are some differences between alternate branches, with ATA+ fibroblasts finding terminal expression at cluster 3, whereas in ARA+ this trajectory terminally ends at cluster 5. Cluster 3 being defined by high expression of EGR+and CXCL12, whereas cluster 5 has high expression of POSTN and COL11A1, consistent with a mesenchymal fibroblast.^17^

### Ligand-receptor interaction by autoantibody subtype in early dcSSc

**Figure 5:**
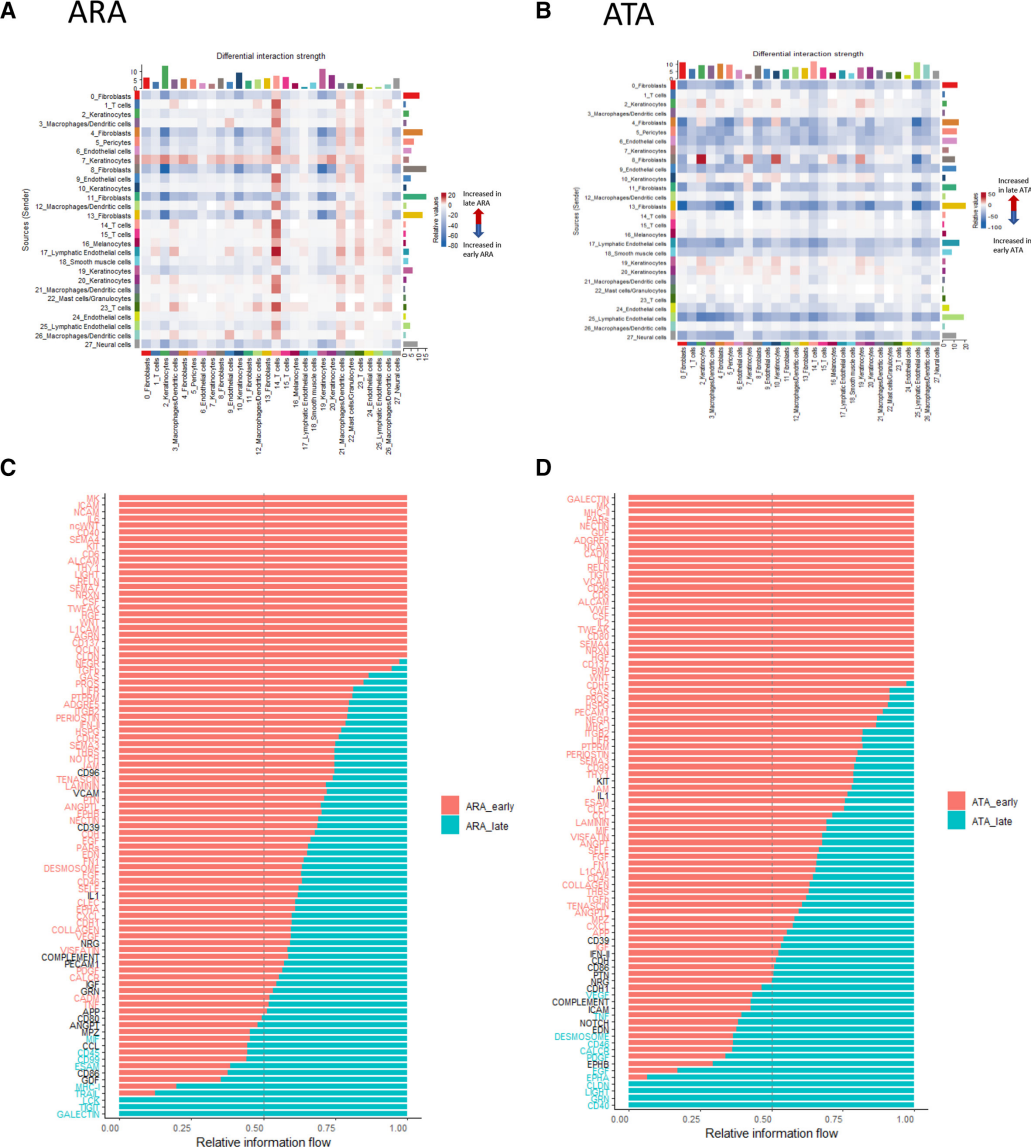
Comparison of signalling strength and pathways between early-stage and late-stage dcSSc by autoantibody subgroup. Heatmap of cell-to-cell interaction differences between early-stage and late-stage disease in (A) ARA+ patients and (B) ATA+ patients. The y axis indicates sending cells, and x axis is receiver cells. Red indicates a stronger signal in late-stage disease, and blue is stronger signal in early disease. (C+D) Differential gene set pathway analysis between early-stage and late-stage disease in (C) ARA+dcSSc and (D) ATA+ patients. Red indicates relative expression in early dcSSc, whereas turquoise is expression from late dcSSc. ARA, anti-RNA-polymerase III; ATA, antitopoisomerase antibody; dcSSc, diffuse cutaneous systemic sclerosis.

Given the differences in gene expression by disease stage and autoantibody subtype, we next focused on how intercellular interactions might differ between stages of the disease. Within ARA patients, (figure 5A), there was uniform upregulation of fibroblast interaction particularly with keratinocytes in early-stage compared with late-stage disease. Notably, one keratinocyte cluster showed upregulation of its interaction with all cell populations, whereas one T cell cluster, seemed to receive increased signal from many cell types in the later stage of the disease. Conversely, in ATA patients, there was much more apparent downregulation of interactions in later stages disease compared with early across not only in fibroblast clusters, but also pericytes, endothelial cells and lymphatic endothelial cells, not seen in ARA patients (figure 5B).

### Biological pathway analysis across disease stage and autoantibody subgroup

Focusing on key biological pathways across all cells, there were many similarities in biological pathway elevation between early-stage and late-stage disease irrespective of antibody (figure 5C,D). This included known pathways and fibroblast markers associated with SSc such as IL6, TGFβ and THY1, thus suggesting a shared SSc signature which is active in the early stages of the disease and diminishes with time. However, it is notable when comparing autoantibody subgroups, that some biological pathways had increased expression in late SSc for one antibody subtype, but were overexpressed in early SSc in the other. For example, in the ATA subgroup, genes associated with VEGF pathways, PDGF and TNF are increased in later stages of disease compared with early stage. However, these same pathways are overexpressed in the early ARA subgroup when compared with the late-stage ARA subgroup. Conversely, genes associated with MHC1, Galectin and CD45 were upregulated in the late-stage ARA subgroup compared with early, whereas in the ATA subgroup, they showed increased expression in the early subgroup compared with the late.

**Figure 6:**
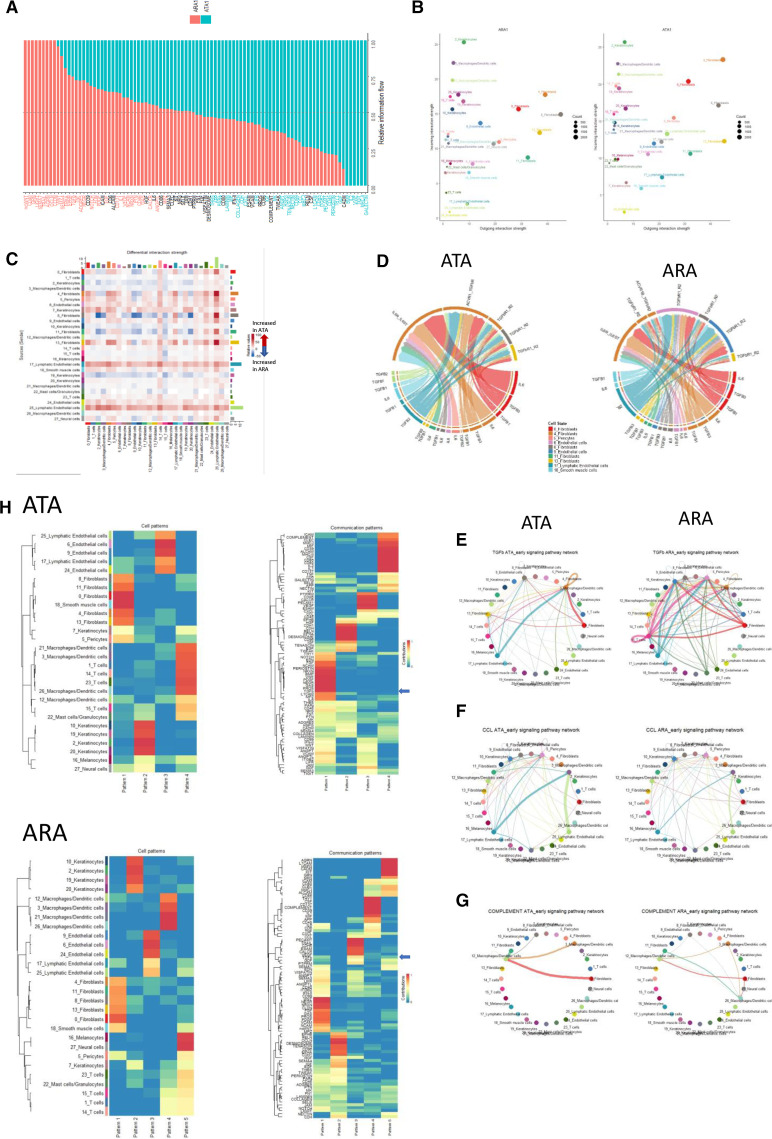
Functional differences between whole skin ARA+ and ATA+ cell cluster interactions for candidate signalling pathways (TGFb, CCL and complement). (A) Relative pathway differential expression in ARA+ early dcSSc (red) and ATA+ early dcSSc (turquoise). (B) Outgoing and incoming signal between cell clusters in whole skin in ARA+ early dcSSc and ATA+ early dcSSc. Notable incoming signal differences seen between fibroblast clusters and lymphatic endothelial cells. (C) Heatmap highlighting differential cell interaction strength between ATA+ and ARA+ early dcSSc. Red indicates higher interaction strength in ATA+ early dcSSc, and blue indicates higher interaction strength in ARA+ early dcSSc. (D) concentrating on TGFβ ligand-receptor interactions, differences can be seen between ATA+ and ARA+ patients in both source of ligands, and more notably where receptors found. In gene expression data from ATA+ skin, receptors were expressed by three fibroblast clusters, whereas in early ARA+ patients, endothelial cells express receptors for the ligands. Plots showing cell to cell interaction and strength of that interaction for specific pathways including (E) TGFβ, (F) CCl signalling and (G) complement by different early antibody states. The strength of the signal is determined by the intercellular lines thickness; the thicker the line, the stronger the signal intensity. (H) Hierarchical pattern of similar expression and cells responding to each pattern of pathway response. In top panels, TGFβ pattern is grouped in pattern 1, and the cells responding to this pathway are predominantly the fibroblasts. In ARA+ early dcSSc, TGFβ expression is grouped in pattern 3, and the cells showing strongest response to pattern 3 are the endothelial and lymphatic endothelial cells. ARA, anti-RNA-polymerase III; ATA, antitopoisomerase antibody; dcSSc, diffuse cutaneous systemic sclerosis.

To dissect some of these pathway differences, a direct comparison of early ARA and early ATA patients was carried out (figure 6A). The most overexpressed pathways in ATA compared with ARA patients included TGFβ, NOTCH signalling, and CD40 LIGHT and TRAIL. Whereas expression for ECM genes including collagen, tenascin and fibrotic pathways such as THBS and THY1 were greater in the early ARA patients.

Further interrogation asked which cells might drive these differences. Within early ATA patients, there are both stronger outgoing and incoming signals from fibroblasts, as well as lymphatic endothelial cells and endothelial cells compared with ARA patients (figure 6B). This is confirmed in figure 6C, which illustrates that most of the interaction between source and receiver cells was greater in the ATA cells compared with the ARA cells. Only one fibroblast population (cluster 8, with high expression of SFRP2 and COMP, therefore, corresponds to cluster 4 in fibroblast subsets) had greater interaction strength with keratinocytes and endothelial cells in the ARA subgroups. Where interaction strength was greater in the ATA patients, signal originates from fibroblasts and lymphatic endothelial cells to fibroblasts, endothelial cells, pericytes and lymphatic endothelial cells.

This difference is further exemplified in other cell signalling interactions (figure 6E–G) such as CCL interactions (a pathway with stronger interactions in the early ATA patients than ARA patients, including CCL2, CCL5 and CCL19, previously shown as key ligand-receptor interactions), and complement, where differing intensity of interaction, as well as unique interactions are seen between the differing autoantibody subtype in early dcSSc patients.

Using hierarchical clustering of these gene sets, we can group not only patterns of gene sets, but also identify which cells respond to these specific gene sets (‘pattern’). Thus, specific cell clusters with similar response to expression of pathways can be grouped with other cells responding to the same gene sets. Focusing on the TGFβ pathway genes, within ATA patients, there is clustering within a ‘pattern module’ which mainly exhibits greatest effect on fibroblasts. However, within the ARA patients, TGFβ clusters within a gene set which mainly exerts effect on endothelial cells (pattern module 3, figure 6H).

## Discussion

Application of scRNAseq allows deconstruction of results obtained using bulk RNA sequencing to explore fibroblast heterogeneity and subsets. This is important because elucidating the cellular basis for heterogeneity in skin severity, natural history and treatment response will help to understand complex pathobiology in SSc and other fibrotic diseases. Both bulk and scRNAseq have provided valuable insights into the molecular changes occurring in parallel to clinical changes in dcSSc. Intrinsic molecular subsets of SSc have been defined based on RNA expression that appears stable over 12 months.^8 18^ However, over longer periods of time, there are changes in gene expression, moving the inflammatory intrinsic subsets towards fibroproliferative or normal-like phenotype.^8 19 20^ Molecular stratification of SSc patients and relationship to clinical phenotype and therapeutic response have previously been explored in these intrinsic subsets.^21–24^

Single-cell analysis has allowed for an increased understanding of the heterogeneity within fibroblast populations.^17 25 26^ It is already appreciated that there are age-related loss of fibroblast priming in healthy skin.^27^ More recently scRNAseq has highlighted population differences between dcSSc, lcSSc and HC, where a population of fibroblasts expressing LGR5 were most abundant in HC, and least in dcSSc.^26^ The same study also demonstrated proportion abundance differences between some fibroblast populations in dcSSc by disease duration. However, autoantibody status was not considered. By analogy, in our cohort, cluster 1 (MGST1+CCN5 FBs) had the highest expression of LGR5 in HCs, and was also the least abundant in the early stages of dcSSc, whereas later stage dcSSc, and HCs showed similar abundance.

The 10 skin fibroblast clusters we identify align with other recent reports. Previous work by Deng *et al* redescribed fibroblast clusters into proinflammatory, mesenchymal, secretory and secretory papillary within fibrotic disease and keloid.^17^ Using their markers, we were able to identify the proinflammatory fibroblasts as clusters 2, 3 and 6, mesenchymal fibroblasts as cluster 5, secretory fibroblasts as cluster 0 and 7, and secretory papillary fibroblasts as cluster 4. They found the mesenchymal fibroblast population to be expanded in keloid and SSc compared with normal scar related fibroblasts. Within our cohort, cluster 5 was identified as POSTN+CCN5- expression. We found this cluster to be expanded in SSc, particularly ARA+ early patients, and ATA+ late-stage patients, and a notable lower abundance in the HC cohort. Other work on SSc fibroblast populations have shown that myofibroblasts tend to have high expression of SFRP4, and ACTA2, a population which resembles cluster 8 of our fibroblast populations.^16^ This same group also identified two major fibroblast populations, with numerous subsidiaries within human skin. They characterised these major fibroblast populations as those expressing SFRP2/DPP4 (consistent with clusters 0, 4 and 7 in our analysis) and those expressing FMO1/LSP1 (clusters 3 and 6).^25^ Within the SFRP2/DPP4 clusters, they further subdivided clusters into those with high expression of WIF1 and NKD2 or PCOLCE and CD55. Our fibroblasts subsets showed consistent gene expression, with cluster 4 having high expression of WIF1 and NKD2, whereas CD55 and PCOLCE were overexpressed in clusters 0 and 7. Due to low expression, we were unable to identify all their smaller clusters within our population, however, they also identified a population of COL11A1 cells, termed dermal sheath cells, which is the marker used by Deng *et al* to identify the mesenchymal fibroblasts.

The papillary fibroblast cluster (fibroblast 4) was noted to have differing interactive strength between autoantibody groups by stage of disease. In the ARA subgroup, there was increased strength interaction with one keratinocyte cluster at late-stage dcSSc. However, within the ATA subgroup, there was no change in interaction strength between papillary fibroblasts and keratinocytes with increased disease duration.

Pseudotime analysis by autoantibody subtype, and cell-to-cell interactions explored in our study highlight key differences in fibroblast trajectories and signalling interaction by autoantibody subtype. Given that this is a treated population, some of the differences may be the result of potential differences in treatment response to mycophenolate mofetil, which all our early dcSSc were receiving, in line with current standards of care.

Differences in TGFβ signal response between ATA and ARA positive early dcSSc patients may help understand recent findings in clinical trials of targeted biological therapy. For example, phase II and phase III trials of tocilizumab (anti-IL6)^28 29^ suggested greater treatment benefit for ATA subjects compared with other autoantibody groups.^30 31^ Transcriptional studies on skin fibroblasts collected during the trial demonstrated that the TGFβ activation signature was almost completely reversed following 6 months of tocilizumab, with numerically greater effect in ATA positive patients.^32^ In this study, we demonstrate that the source of the TGFβ signal originates from the fibroblasts, endothelial cells and smooth muscle cells. However, the ligand-receptor interaction was different by autoantibody, with receptors predominantly being found on the fibroblast clusters in ATA patients, whereas in ARA patients, the receptors were mainly on fibroblasts, endothelial and lymphatic endothelial cells. Our data show there is a diminished response to TGFβ signalling in ARA+ patients compared with ATA, thus suggesting that the impact on fibroblasts through blockade of this pathway will also be clinically less significant.

The strengths of this study are that we recruited well defined patient subgroups to draw our conclusions. By focusing on stage of disease and differing autoantibodies, we could explore differences between these autoantibody subgroups over time, something which has not been possible over shorter time intervals in a 12-month prospective study design. Our patients are from a well-established and characterised observational cohort, with current standard of care treatment in accordance with treatment recommendations. Thus all patients had received MMF and other supportive therapy meaning that observed cellular differences are less likely be directly due to the treatment itself, but may reflect contrasting disease biology across disease stage and ANA subgroup.

Limitations of the study include the small sample size in each patient subgroup. This is also a real-world population and so there may be greater clinical variability than in a prospective clinical trial cohort. However, recent analysis has confirmed the similarity in prognostic markers between our real world cohort and patients in a relevant prospective trial.^30^ Age differences between subjects may also be a limitation. It is appreciated that there is loss of fibroblast priming with age, however, given our established cohort and early-stage cohort are similar in age by autoantibody, and age-related changes should be comparable between the autoantibody groups.

In conclusion, we demonstrate key cellular differences, particularly within fibroblasts, between patients with dcSSc based on autoantibody and stage of disease. Appreciating these differences will help better understand the pathobiological basis for clinical diversity in SSc. Our findings have important implications for trial design, including future targeted therapeutics, to ensure that most informative patients are recruited into early-stage clinical trials.

## Data Availability

Data are available on reasonable request. The data are available for the purposes of academic research on reasonable request to the corresponding author.
