# Supplementary material for: Single-cell analysis reveals key differences between early-stage and late-stage systemic sclerosis skin across autoantibody subgroups
Source: Ann Rheum Dis. 2023 Aug 14;82(12):1568–79. doi: 10.1136/ard-2023-224184 (PMC10646865; doi:10.1136/ard-2023-224184)

Supplementary figures

Supplementary Table 1

	Early ARA	late ARA	early ATA	late ATA	HC
Age (yrs)	56	70	45	60	
Female (%)	3	2	2	2	3
Disease duration (months)	51	133	38	226	
MRSS	22	7	17	11	
Autoantibody					
ARA	3	3	0	0	3
ATA	0	0	3	3	
Immunosuppression					
MMF	3	1	3	3	
MTX	1	0	0	0	
Prednislone <10 mg	0	0	1	2	
Organ complications					
ILD	1	1	2	3	
Myositis	0	0	1	0	
Renal crisis	0	1	0	0	
PAH	0	1	0	0	

Supplementary Figure 1

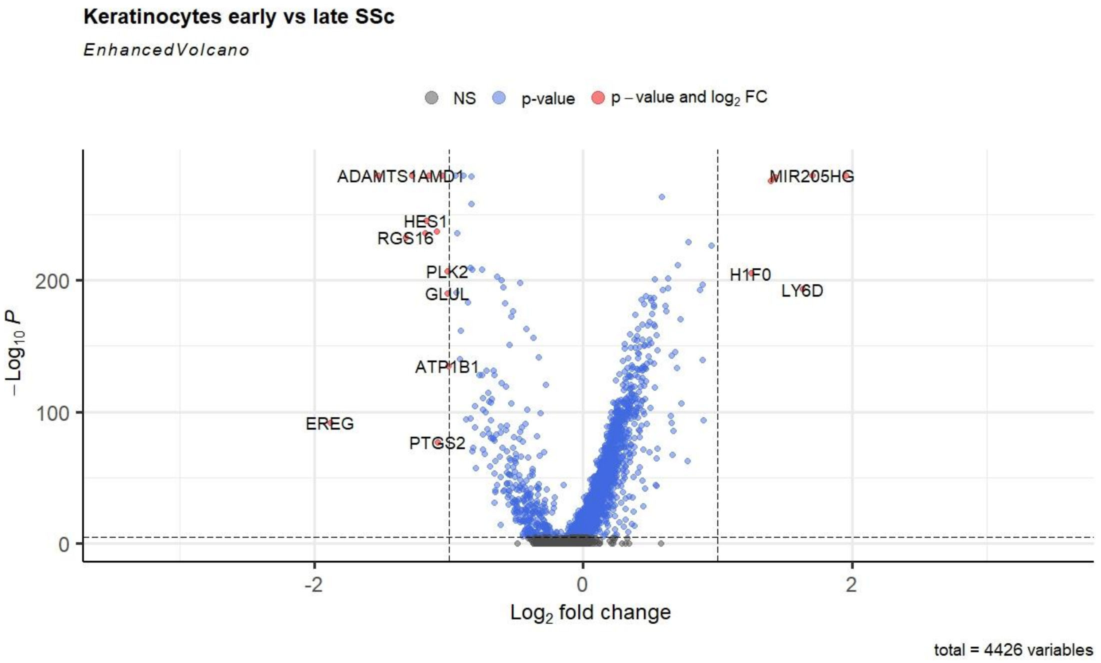

Higher in early dcSSc				Higher in late dcSSc		
gene list	avg_log2FC	p_val_adj		gene list	avg_log2FC	p_val_adj
LGALS7	1.954	<0.0001		EREG	-1.879	<0.0001
MIR205HG	1.706	<0.0001		ADAMTS1	-1.528	<0.0001
LY6D	1.631	<0.0001		RGS16	-1.319	<0.0001
CXCL14	1.429	<0.0001		DNAJA1	-1.277	<0.0001
HIST1H1C	1.395	<0.0001		HSPH1	-1.177	<0.0001
H1F0	1.250	<0.0001		HES1	-1.170	<0.0001
PLIN2	0.950	<0.0001		HEXIM1	-1.148	<0.0001
AKR1C1	0.894	<0.0001		DDX3Y	-1.089	<0.0001
KRT15	0.887	<0.0001		PTGS2	-1.081	<0.0001
KRT5	0.886	<0.0001		AMD1	-1.051	<0.0001

Supplementary Figure 2

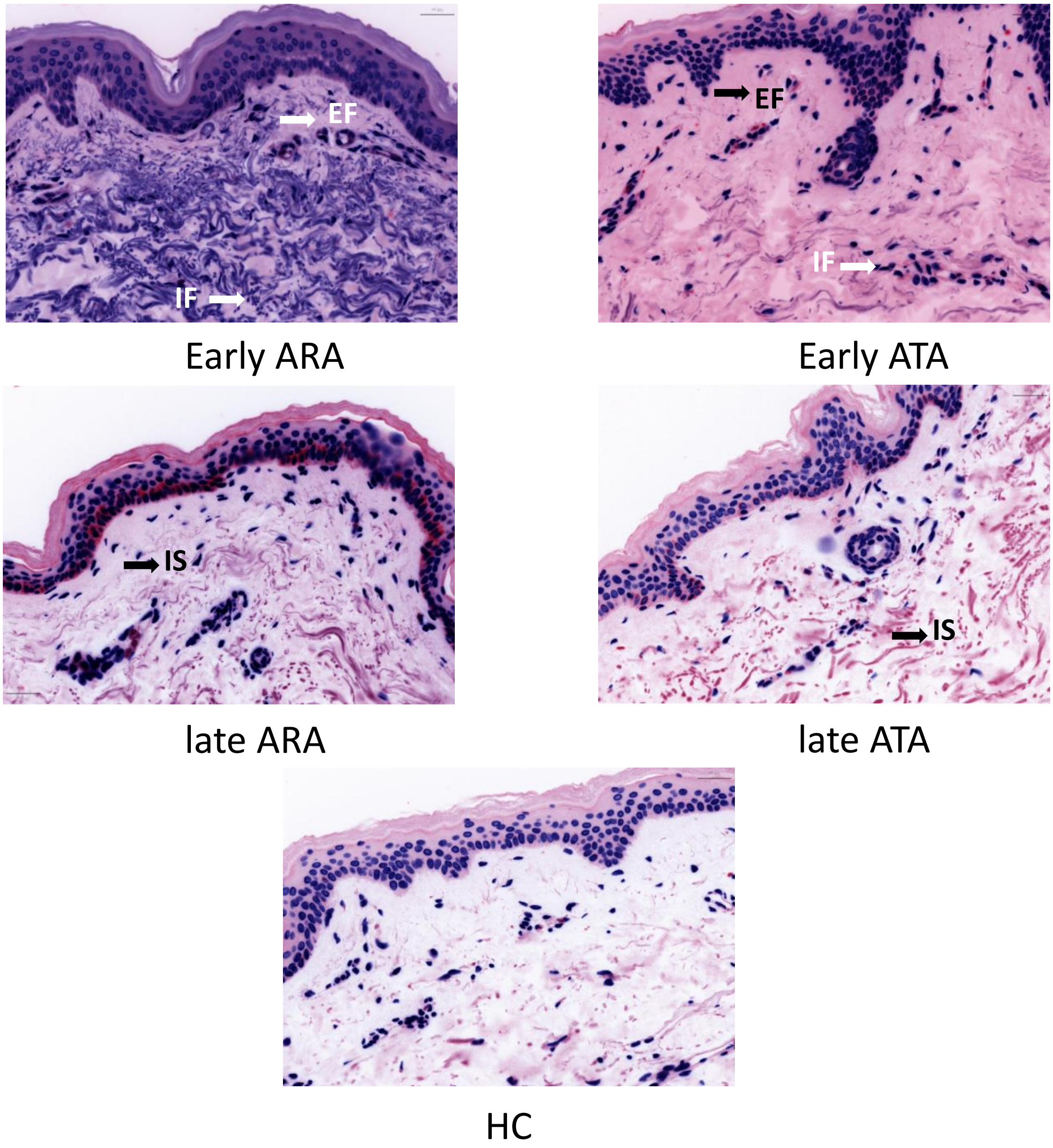

Supplementary Figure 3

A

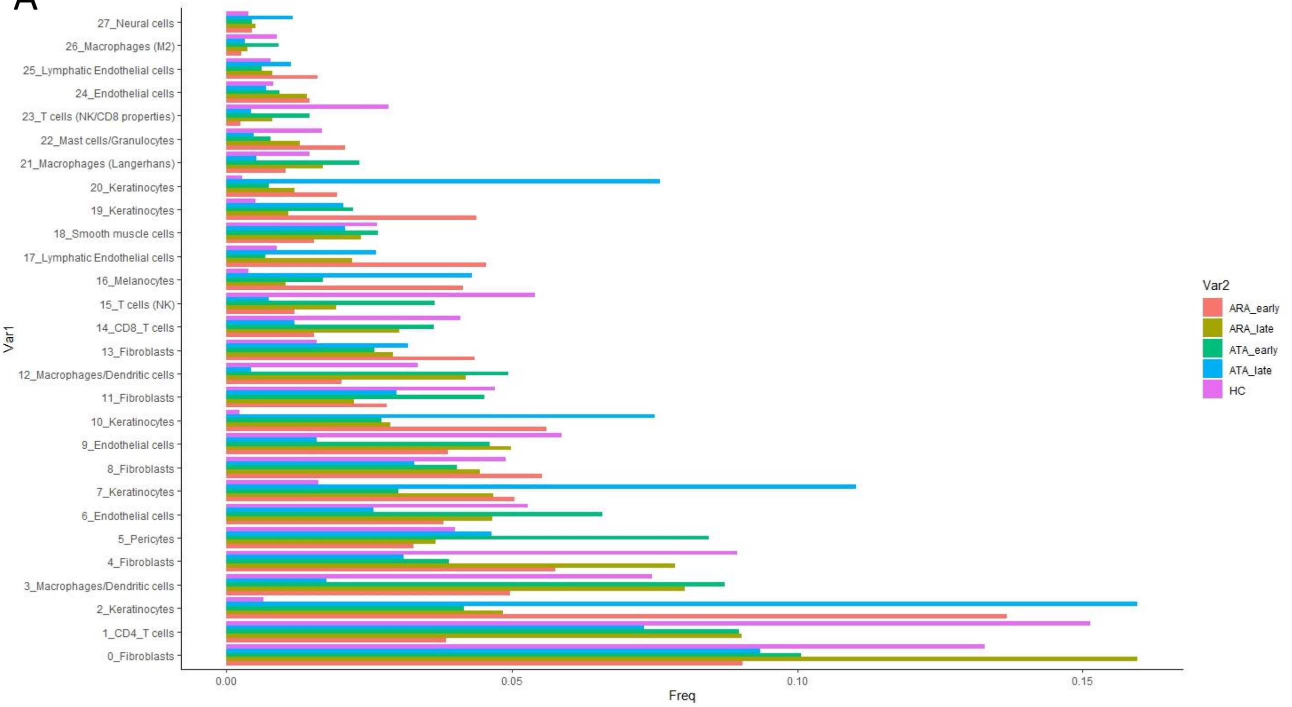

B Macrophages/Dendritic cells

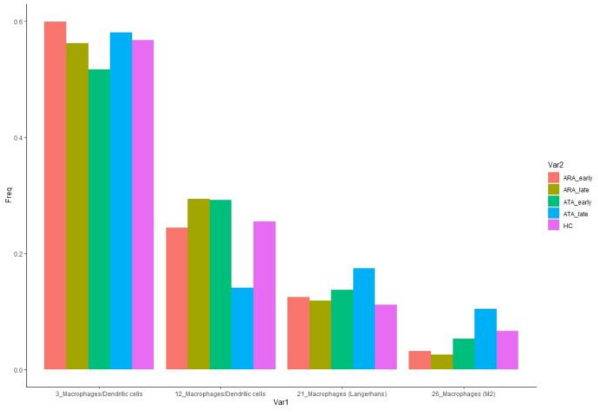

C T Cells

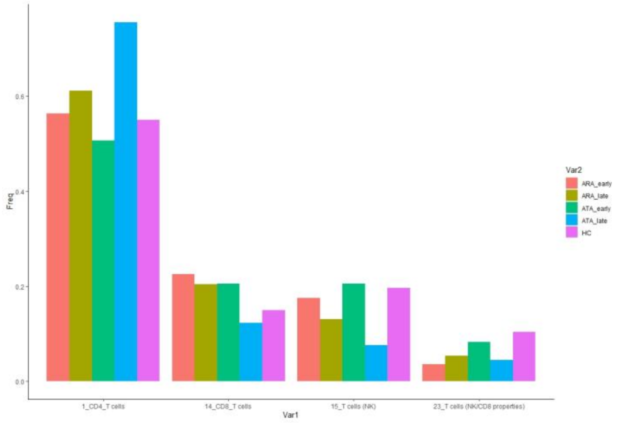

Supplementary figure 4

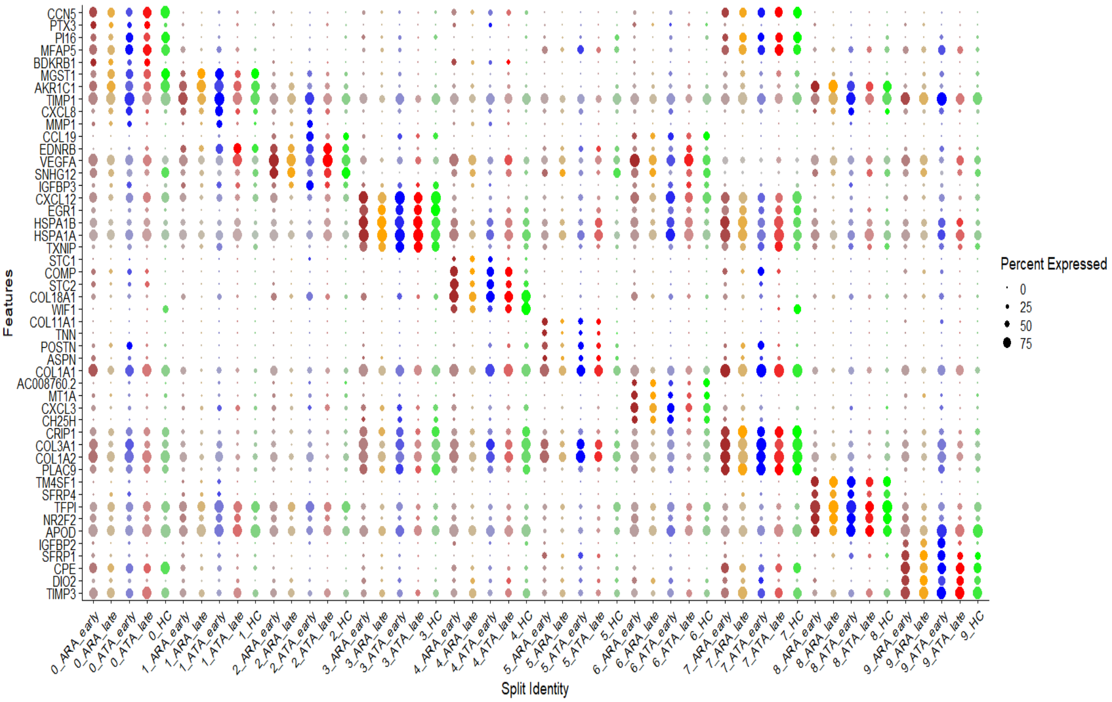

Supplementary figure 5

ARA early vs late

ATA early vs late

ARA early vs ATA early

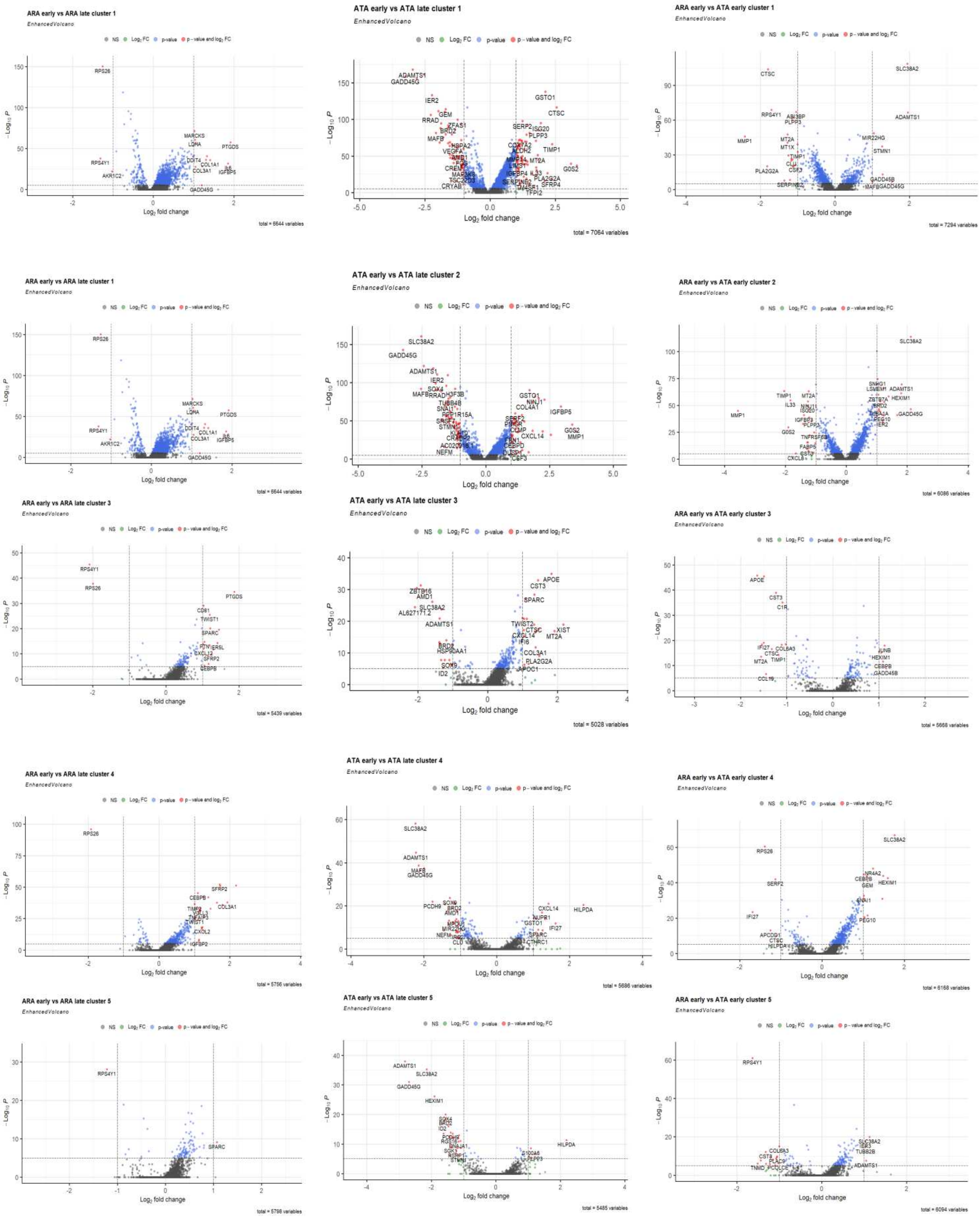

ARA early vs late | ATA early vs late | ARA early vs ATA early

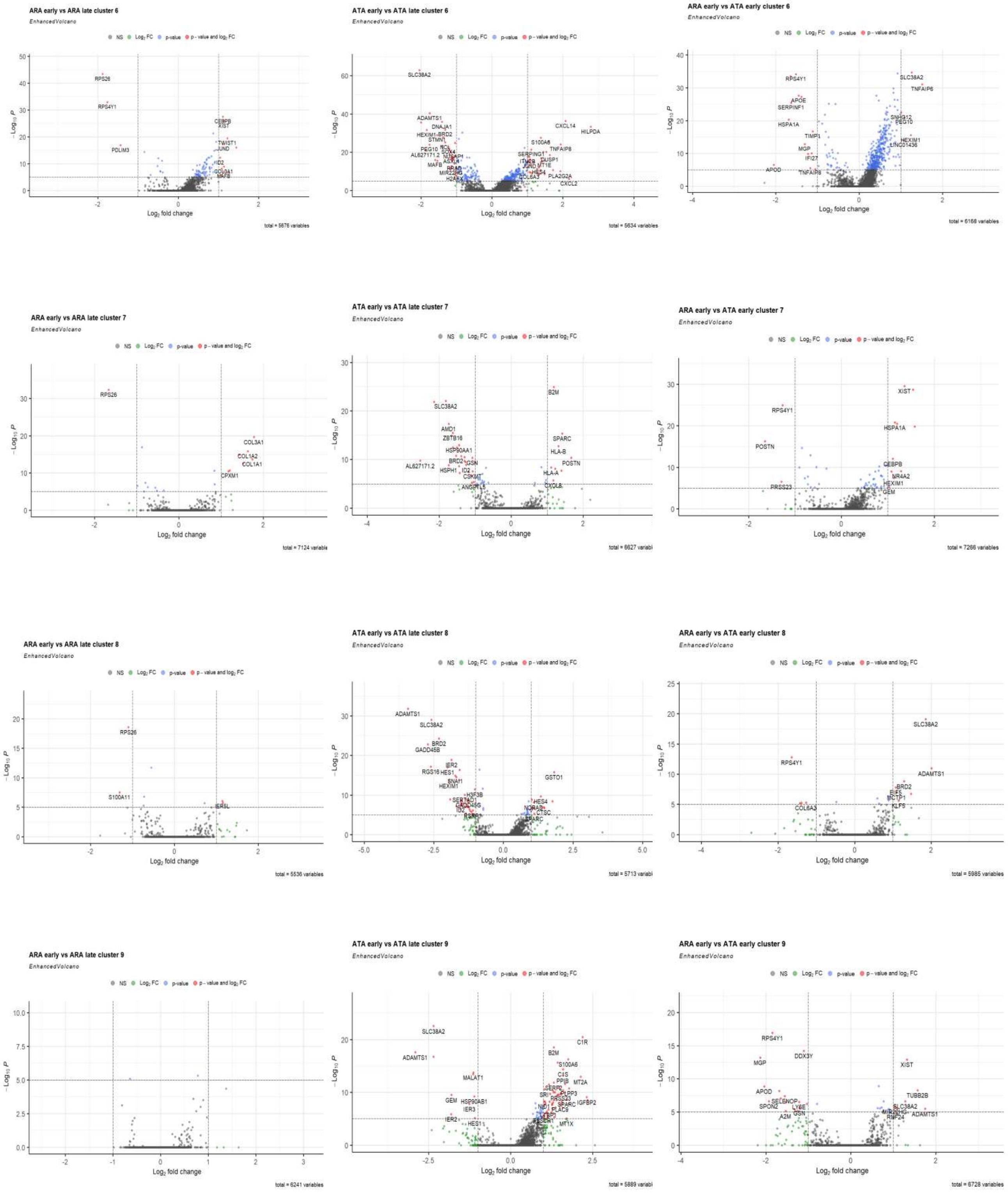

Supplement: Supplementary data [file ard-2023-224184supp001.pdf]
